# Enrollment of reproductive age women in community-based health insurance: An evidence from 2019 Mini Ethiopian Demographic and Health Survey

**DOI:** 10.3389/fpubh.2023.1067773

**Published:** 2023-03-30

**Authors:** Simegnew Handebo, Takele Gezahegn Demie, Berhanu Teshome Woldeamanuel, Tolesa Diriba Biratu, Getachew Tilahun Gessese

**Affiliations:** School of Public Health, St. Paul’s Hospital Millennium Medical College, Addis Ababa, Ethiopia

**Keywords:** community-based health insurance, enrollment, women of reproductive age, demographic and health survey, Ethiopia

## Abstract

**Background:**

Universal health coverage (UHC) is aimed at ensuring that everyone has access to high-quality healthcare without the risk of financial ruin. Community-based health insurance (CBHI) is one of the essential means to achieve the sustainable development goals (SDGs) global health priority of UHC. Thus, this study assessed health insurance enrollment and associated factors among reproductive age women in Ethiopia.

**Methods:**

We computed the health insurance enrollment of reproductive-age women using secondary data from the recent Ethiopian Mini Demographic and Health Surveys (EMDHS) 2019. The EMDHS was a community-based cross-sectional study carried out in Ethiopia from March 21 to June 28, 2019. Cluster sampling with two stages was employed for the survey. The study comprised 8885 (weighted) reproductive-age women. STATA 14 was used for data processing and analysis. Bivariate and multivariable logistic regression analyses were conducted. Adjusted odds ratio (AOR) with 95% confidence interval (CI) was reported and statistical significance was set at a value of *p* < 0.05.

**Results:**

Of the 8,885 study participants, 3,835 (43.2, 95% CI; 42.1, 44.2%) of women had health insurance. Women aged 20–24 years, 25–29 years, and 30–34 years less likely to enroll in health insurance compared to their younger counterparts (15–19 years). Women living in rural areas, had greater than five family sizes, living with a female household head, and having more than five living children were negatively associated with enrollment in health insurance. Besides, health insurance enrollment among reproductive-age women is significantly affected by region and religious variation.

**Conclusion:**

The overall CBHI enrolment among reproductive-age women in Ethiopia was low. To achieve the SDGs of reducing maternal mortality ratio and neonatal mortality, improving reproductive-age women’s access to health insurance is essential. The national, regional, and local officals, policymakers, NGOs, program planners, and other supporting organizations working on improving health insurance enrollment of reproductive age women need to create awareness and support them based on these significant factors.

## Introduction

Healthcare financing is central to the operation of healthcare systems and the achievement of health-related sustainable development goals (SDGs), including universal health coverage (UHC). The healthcare financing policies of a country determine who has access to health services and the level of financial protection provided to the population (1, 2). A large share of the population in low-income countries faces financial barriers to access health services. This has a detrimental impact on the use of services and how quickly they are used. Healthcare expenses are typically paid out-of-pocket at the time of service delivery in the majority of developing nations, which may limit access to healthcare services (3).

In 2015, the year the SDGs were adopted, globally 926.6 million people incurred catastrophic health spending, which means out-of-pocket health expenditure exceeding 10% of the household income, and 208.7 million people incurred out-of-pocket health expenses surpassing 25% of the household income (4). The majority of policy discussions center on preventing the poor from sliding into a poverty trap that is often caused by medical expenses (5). Policy-makers and the health system of many countries, particularly in sub-Saharan African countries, developed community-based health financing (CBHF) schemes to address this problem. CBHF has piqued the interest of governments in the region, which are caught between the opposing pressures of tight public healthcare budgets and the population’s limited ability to pay for healthcare (6, 7).

Community-based health insurance (CBHI) programs typically consist of voluntary, nonprofit health insurance plans wherein local residents pool funds to offset the cost of healthcare. It is a type of micro health insurance, a general term for health insurance aimed at those with lowest incomes. The specific feature of CBHI is the community involvement in driving its setup and management. Despite the fact that CBHI programs vary in their conception and execution, they are always founded on the idea of risk sharing and entail routine payments of a small premium in exchange for a reduction in direct payments at the point of services (7, 8).

Direct payment at the point of services has been found to delay or dissuade the utilization of healthcare (7, 9). In contrast, studies demonstrate the positive effect of CBHI in protecting the poor, reducing financial barriers to care, and improving the use of priority health services (5, 6, 10). CBHI contributes to improving access to outpatient and inpatient care (11). Demissie and Negeri also reported that CBHI was linked to higher outpatient care utilization (12).

Health insurance of reproductive age women is crucial to ensure access to healthcare, promote treatment adherence, protect against financial burdens of care, and supports self-reported health and well-being. It facilitates access to interventions to minimize behavioral risk factors, manage chronic diseases, support preconception healthcare, and access to diagnosis and treatment pregnancy-related physical and mental health conditions, which is associated with improved maternal and infant health outcomes (13–15). It also expands access and use of contraception services, which minimizes unintended pregnancies and improves pregnancy spacing (13, 14, 16).

In Indonesia, 40.0% of women were covered by health insurance (17). However, in sub-Saharan African countries, the health insurance enrollment among reproductive-age women is unacceptably low (8.5%) (18). Similarly, 7.56, 7, 4.7%, and only less than 3%, of women in East African countries, Kenya, Ethiopia, and Nigeria enrolled in health insurance, respectively (15, 18–21). Due to this health-service delivery and health status of the reproductive age women remained low mainly (18). Age, educational status, employment status, marital status, place of residence, wealth index, media exposure, geo-political zone, residing in a female-headed household, visiting health facility within 12 months and being visited by field workers are statistically significant predictors of CBHI enrolment (15, 18–21). Hence, health-care delivery and reproductive-age women’s health status remained primarily low.

In Ethiopia, health service utilization remained low and household out-of-pocket expenditure accounted for more than one-third of total health expenditure (22, 23). Ethiopia has endorsed two risk-pooling arrangements namely CBHI and Social Health Insurance (22, 24, 25). In June 2011, the Government of Ethiopia launched a pilot CBHI scheme in 13 rural districts located in four main regions (Tigray, Amhara, Oromia and SNNPR) of the country. CBHI uptake was so remarkable in the piloting year which was raised to 41% in the first year and 48% in the second year. Although scheme enrollment has increased generally over the past 2 years, there is a significant churn rate, with 18% of households who participated in the first year ceasing to make payments in the second year (26, 27). Both inpatient and outpatient healthcare services in public facilities are covered under the program. With this scheme, using healthcare from private providers is forbidden unless a service or drug is unavailable at a public facility (26). Ethiopia launched a CBHI scheme in 2011 with a vision of reaching 80% of all districts and 80% of its population by 2020. The CBHI program aims to alleviate the devastating out-of-pocket medical expenses that affect the 85% of Ethiopians. The goals of CBHI include reducing catastrophic out-of-pocket costs, lowering financial obstacles, increasing the use of health services, enhancing quality of care, enhancing health equity, and enhancing sustainable health finance through mobilization of domestic resources (28, 29).

Women and children in particular are expected to be benefited from this system without asking the male head of household for money (30). To do so, women should be enrolled in the CBHI scheme and own the membership card. Previous studies in the country were conducted on a small scale with a small sample size and a restricted geographic location. Using the 2016 EDHS data at the national level Kebede et al. analyzed geographic variation of health insurance coverage among women in Ethiopia (31), while Weldesenbet et al. assessed health insurance coverage in East Africa and associated factors (19). Besides, there is a lack of recent nationwide evidences on health insurance among Ethiopian women of reproductive age and the factors that affect it. So, the current study aimed to assess the enrollment of health insurance and its associated factors among reproductive-age women in Ethiopia using the current 2019 EMDHS data. This finding provides evidence for policymakers, health planners, and programmers working on health insurance-related interventions geared towards achieving UHC and improving financial protection of reproductive-age women and their children.

## Methods

### Study design and settings

The second from 2019 was used in this study. A cross-sectional nationwide study centered in communities was carried out from March 21 to June 28, 2019. Ethiopia is divided into nine regional states on an administrative level.

The study used the 2019 EMDHS dataset, which is the second mini Demographic and Health Survey (DHS) in Ethiopia. It is a national wide community-based cross-sectional study carried out from March 21 to June 28, 2019. Ethiopia is structured into nine regional states on an administrative level (Tigray, Afar, Amhara, Oromia, Somali, Benishangul-Gumuz, Southern Nations Nationalities and People (SNNP) (incorporate the newly formed Sidama and Southwest Ethiopia regions), Gambela, and Harari) and two city administrations (Addis Ababa and Dire Dawa). The country is the second-most populous country in Africa, is situated in the Horn of Africa between 30° and 15° North latitude and 33°–48° East longitude.

### Study population and sampling procedures

As a sampling frame, the survey employed a comprehensive list of the 149,093 enumeration areas (EAs) produced for the Ethiopia Population and Housing Census (EPHC). The census frame is a complete list of the 149,093 EAs created for the 2019 EPHC. An EA is a geographic area that typically has an average of 131 households. Information regarding the EA location, type of residence (urban or rural), and an estimated number of residential households are included in the sampling frame (32).

A two-stage cluster sampling technique was employed to select the study participants. A total of 305 EAs — 93 in urban and 212 in rural areas—were chosen in the first round, with probability proportional to EA size. In the second stage, a set number of 30 households per cluster were selected with an equal probability systematic selection from the newly created household listing. All women aged 15–49 who were either permanent residents of the selected households or guests who stayed there the night before the survey were eligible to participated in the study. In EMDHS, 5,753 children aged 0–59 months, 8,855 reproductive-age women (age 15–49), and 40,659 household members in selected 8,663 households were included. From 9,012 eligible women identified for the interview, 8,885 women completed the interviewyielding a response rate of 99% (32). All the reproductive-age women living in nine regions (Tigray, Afar, Amhara, Oromia, Somali, Benishangul-Gumuz, Southern Nations Nationalities and People (SNNP) (incorporate the newly formed Sidama and Southwest Ethiopia regions), Gambela, and Harari) and two administrative cities (Addis Ababa and Dire Dawa) of Ethiopia were included in the study.

### Data source

The data source for this study was the EMDHS survey, which was collected in 2019. EDHS is collected every 5 years by the Ethiopian Central Statistical Agency (CSA) along with ICF International. The dataset was obtained from the Measure DHS website^1^ in STATA format after permission was obtained. The outcome and explanatory variables were extracted from the EMDHS household and individual data set.

### Study variables

#### Dependent variable

The outcome variable of the study was CBHI enrollment among reproductive-age women. In the study, women were asked the question that says; “Is your household enrolled in a Community-Based Health Insurance scheme?” and the responses were coded as 1 if “yes” and 0 if “no.” The used the answer to this query to determine the percentage of women in reproductive age who are enrolled in community-based insurance.

#### Independent variables

The independent variables were maternal age, educational status, marital status, religion, region, wealth index, place of residence, family size, literacy, sex of head of households, age of household head, births in the last 5 years, total children ever born, number of living children, age of respondent at first birth, and number of under-five children in the household.

### Data management and analysis

The STATA 14 statistical software was used to extract, clean, and analyze the data. To account for the unequal probability of strata selection and the non-response rate among study participants, sample weights were used. The EMDHS methodology report provides a thorough explanation of the weighting process (32). A descriptive analysis was conducted to display the sociodemographic distribution of participants and the proportion of women enrolled in the CBHI. Bivariate and multivariable logistic regression were employed to identify the factors associated with enrolment in CBHI. As potential variables for the multivariable logistic regression analysis, variables with a value of p of less than 0.2 in the bivariate analysis were chosen. To evaluate the strength of association between the outcome and the independent variables, crude and adjusted odds ratios (AOR) with corresponding 95% confidence intervals (CI) were also employed. The variance inflation factors (VIF) was used to examine the multi-collinearity and the value of each variable was less than five. The threshold for statistical significance was set at a value of *p* < 0.05. The models fitness was checked by the Hosmer and Lemeshow test of goodness of fit and the data were fitted to the model (value of *p* = 0.246).

## Results

### Socio-demographic characteristics

A total of 8,885 women participated in the study yielding a response rate of 99%. Nearly one-fourth (24.87%) of women were aged 15–19 years old and 10.98% of them were insured. More than half (66.0%) of respondents were married and 28.5% were insured. Of the subjects, 21.1 and 14.8% of women living in the Oromia and Amhara regions had CBHI, respectively. Of the 41.7% women who had completed primary school and 18.1% of them had CBHI. More than three-fourths (79.4%) of households were headed by males and of those, 34.85% of women were insured. Concerning literacy status, half (50.29%) of women were unable to read at all and 20.97% of them had CBHI. Of 25.74% women in the richest wealth quartile, 13.67% were insured. Four thousand five hundred and twenty-two (50.9%) of study participants had greater and equal to five family size and of those 24.31% of them had CBHI (Table 1).

**Table 1:** Sociodemographic characteristics of reproductive age women in Ethiopia, 2019 (*n* = 8885).

Variables	Description	Health insurance (weighted)		
		No, *n* (%)	Yes, *n* (%)	Total, *n* (%)
Age	15–19	1,235 (13.89)	976 (10.98)	2,210 (24.87)
	20–24	826 (9.3)	654 (7.37)	1,481 (16.66)
	25–29	1,000 (11.25)	668 (7.51)	1,667 (18.77)
	30–34	688 (7.74)	472 (5.31)	1,160 (13.05)
	35–39	604 (6.8)	461 (5.18)	1,065 (11.99)
	40–44	402 (4.52)	338 (3.8)	739 (8.32)
	45–49	296 (3.33)	267 (3.01)	563 (6.34)
Marital status	Never married	1,342 (15.10)	984 (11.07)	2,325 (26.17)
	Married	3,330 (37.48)	2,534 (28.52)	5,864 (66)
	Separated	379 (4.26)	317 (3.57)	696 (7.83)
Place of residence	Urban	1,476 (16.61)	1,386 (15.60)	2,861 (32.21)
	Rural	3,575 (40.23)	2,449 (27.56)	6,024 (67.8)
Region	Tigray	482 (5.42)	147 (1.65)	629 (7.08)
	Afar	38 (0.43)	48 (0.54)	85 (0.96)
	Amhara	710 (7.99)	1,316 (14.81)	2026 (22.80)
	Oromia	1,469 (16.53)	1878(21.14)	3,347 (37.67)
	Somali	403 (4.54)	17 (0.19)	420 (4.73)
	Benishangul	95 (1.07)	3 (0.04)	98(1.11)
	SNNPR	1,672 (18.82)	33 (0.37)	1705 (19.19)
	Gambela	38 (0.43)	2 (0.03)	40 (0.46)
	Harari	19 (0.22)	8 (0.09)	27 (0.30)
	Addis Ababa	112 (1.26)	330 (3.71)	442 (4.97)
	Dire Dawa	12 (0.13)	53 (0.59)	64 (0.73)
Educational level	No education	2,117 (23.83)	1,472 (16.57)	3,589 (40.40)
	Primary	2091 (23.53)	1,610 (18.12)	3,701 (41.65)
	Secondary	599(6.74)	489 (5.51)	1,088 (12.25)
	Higher	244 (2.74)	263 (2.96)	507 (5.70)
Religions	Orthodox	1790 (20.15)	1894 (21.32)	3,685 (41.47)
	Protestant	1778 (20.01)	657 (7.4)	2,435 (27.41)
	Muslim	1,390 (15.64)	1,230 (13.84)	2,619 (29.48)
	Other^&^	92 (1.03)	54 (0.61)	145 (1.64)
Sex of household head	Male	3,953 (44.5)	3,097 (34.85)	7,050 (79.35)
	Female	1,097 (12.34)	738 (8.31)	1835 (20.65)
Literacy	Able to read whole sentence	1,494 (16.81)	1,487 (16.73)	2,981 (33.55)
	Able to read parts of sentence	780 (8.78)	472 (5.31)	1,252 (14.09)
	Cannot read at all	2,605 (29.32)	1863 (20.97)	4,468 (50.29)
	Not assessed	171 (1.93)	13 (0.14)	184 (2.07)
Wealth index	Poorest	945 (10.63)	492 (5.54)	1,437 (16.17)
	Poorer	900 (10.13)	715 (8.05)	1,615 (18.18)
	Middle	1,006 (11.32)	665 (7.49)	1,671 (18.81)
	Richer	1,127 (12.68)	747 (8.41)	1874 (21.1)
	Richest	1,072 (12.07)	1,215 (13.67)	2,287 (25.74)
Births in last 5 years	0	2,761 (31.07)	2,197 (24.73)	4,958 (55.81)
	1	1,402 (15.78)	1,104 (12.42)	2,506 (28.20)
	2+	887 (9.98)	534 (6.01)	1,421 (15.99)
Family size	≤ 5	2,363 (26.59)	2,160 (24.31)	4,522 (50.90)
	> 5	2,688 (30.25)	1,675 (18.85)	4,363 (49.10)
Age of household head	≤ 24	279 (3.14)	264 (2.97)	543 (6.11)
	25–39	1892 (21.30)	1,385 (15.58)	3,277 (36.88)
	40–54	1857 (20.90)	1,360 (15.31)	3,217 (36.21)
	≥ 55	1,021 (11.50)	826 (9.30)	1848 (20.8)
Number of under 5 children in household	≤ 2	4,816 (54.21)	3,666 (41.26)	8,483 (95.47)
	2 to 5	234 (2.63)	168 (1.9)	402 (4.53)
Number of living children	≤ 2	2,888 (32.51)	2,384 (26.83)	5,272 (59.34)
	2 to 5	1,337 (15.04)	971 (10.92)	2,307 (25.97)
	>5	825 (9.29)	480 (5.4)	1,305 (14.69)

SNNPR, Southern Nations, Nationalities, and Peoples’ Region; &, catholic, traditional, and others.

### Community-based health insurance enrolment

**Figure 1:**
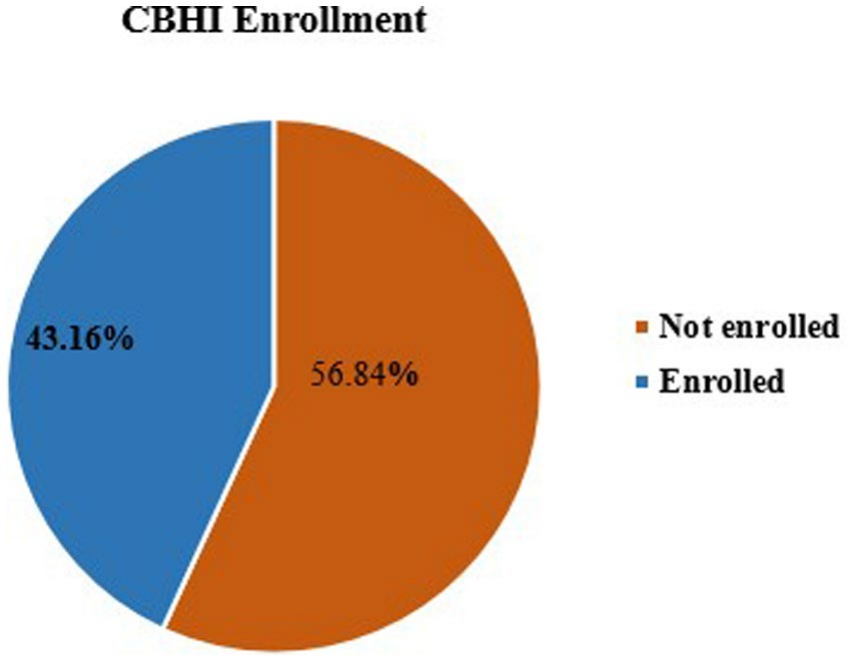
Prevalence of enrolment in community-based health insurance among reproductive-age women, 2019.

As illustrated in Figure 1 below, the overall prevalence of CBHI enrollment among reproductive-age women in Ethiopia was 43.2% (95% CI; 42.1, 44.2%).

### Factors associated with CBHI enrolment

Eleven variables with a value of p of less than 0.2 in the bivariable analysis were taken into account in the multivariable analysis model. In multivariable binary logistic regression analysis, women who aged 20–24 years (AOR = 0.77, 95% CI: 0.65, 0.92), 25–29 years (AOR = 0.70, 95% CI: 0.57, 0.85) and 30–34 years (AOR = 0.74, 95% CI: 0.59, 0.93) were less likely enrolled in the CBHI compared to the younger counterparts (15–19 years). Women living in rural areas were 25% less likely enrolled in CBHI than urban residents (AOR = 0.75, 95% CI: 0.66, 0.85).

Compared to women in the Tigray region, those in Afar region (AOR = 3.94, 95%CI: 2.41, 6.45), Amhara region (AOR = 6.10; 95% CI: 4.94, 7.53), Oromia region (AOR = 4.69, 95%CI: 3.73, 5.89), Addis Ababa (AOR = 8.50; 95%CI: 6.31, 11.45) and Dire Dawa (AOR = 13.05, 95%CI; 6.67, 25.53) had higher odds of enrollment in CBHI. The likelihood of women enrollment in CBHI in the Somali (AOR = 0.15, 95%CI; 0.09, 0.26), Benishangul Gumuz (AOR = 0.10, 95%CI; 0.03, 0.32), SNNPR (AOR = 0.08, 95%CI; 0.05, 0.12), and Gambela (AOR = 0.25; 95%CI; 0.06, 0.96) regions was lower as compared to the Tigray region. Women who had a higher education level were 1.46 times higher enrollment in CBHI than women who had no education (AOR = 1.46, 95%CI; 1.08, 1.98).

Women who were affiliated with the protestant religion were 28% less likely to enroll in CBHI than those affiliated with orthodox religions (AOR = 0.72, 95% CI; 0.60, 0.85). Whereas, women who were affiliated with Muslim (AOR = 1.17; 95% CI; 1.003, 1.35) and other (catholic, traditional, and others) (AOR = 1.86; 95% CI; 1.14, 3.03) religions were more likely enrolled in CBHI compared to those affiliated with Orthodox religion. Having greater than five family size (AOR = 0.84; 95% CI; 0.75, 0.95), female household head (AOR = 0.81; 95% CI; 0.70, 0.94), and having more than five living children (AOR = 0.75; 95% CI; 0.59, 0.95) were negatively associated with enrollment in CBHI (Table 2).

**Table 2:** Multivariable logistic regression of factors associated with CBHI enrolment among reproductive age women in Ethiopia, 2019 (*n* = 8885).

Variables	Description	COR (95% CI)	AOR (95% CI)
Age	15–19	1	1
	20–24	1.002 (0.88, 1.14)	0.77 (0.65, 0.92)**
	25–29	0.85 (0.74, 0.96) *	0.70 (0.57, 0.85)**
	30–34	0.87 (0.75, 1.004)	0.74 (0.59, 0.93)*
	35–39	0.97 (0.83, 1.12)	0.87 (0.68, 1.11)
	40–44	1.06 (0.90, 1.26)	1.08 (0.82, 1.42)
	45–49	1.14 (0.95, 1.37)	1.28 (0.94, 1.74)
Marital Status	Never married	1	1
	Married	1.04 (0.94, 1.14)	1.19 (0.99, 1.43)
	Separated	1.14 (0.96, 1.35)	1.17 (0.92, 1.49)
Place of residences	Urban	1	1
	Rural	0.73 (0.67, 0.80) **	0.75 (0.66, 0.85)**
Region	Tigray	1	1
	Afar	4.13 (2.60, 6.58) **	3.94 (2.41, 6.45)**
	Amhara	6.07 (4.94, 7.46) **	6.10 (4.94, 7.53)**
	Oromia	4.19 (3.44, 5.10) **	4.69 (3.73, 5.89)**
	Somali	0.14 (0.08, 0.23) **	0.15 (0.09, 0.26)**
	Benishangul	0.11 (0.04, 0.34) **	0.10 (0.03, 0.32)**
	SNNPR	0.07 (0.04, 0.10) **	0.08 (0.05, 0.12)**
	Gambela	0.20 (0.05, 0.76) **	0.25 (0.06, 0.96)*
	Harari	1.29 (0.55, 3.05)	1.12 (0.47, 2.68)
	Addis Ababa	9.66 (7.28, 12.82)**	8.50 (6.31, 11.45)**
	Dire Dawa	14.66 (7.59, 28.31)**	13.05 (6.67, 25.53)**
Highest education	No education	1	1
	Primary	1.11 (1.01, 1.22) *	1.10 (0.93, 1.31)
	Secondary	1.18 (1.03, 1.35) *	1.14 (0.90, 1.46)
	Higher	1.55 (1.29, 1.87) **	1.46 (1.08, 1.98)*
Religion	Orthodox	1	1
	Protestant	0.35 (0.31, 0.39)**	0.72 (0.60, 0.85) **
	Muslim	0.84 (0.76, 0.92)**	1.17 (1.003, 1.35)*
	Others^&^	0.56 (0.39, 0.78)**	1.86 (1.14, 3.03) *
Family size	≤ 5	1	1
	> 5	0.68 (0.63, 0.74) **	0.84 (0.75, 0.95)**
Sex of household head	Male	1	1
	Female	0.86 (0.77, 0.95) **	0.81(0.70, 0.94)**
Literacy	Able to read the whole sentence	1	1
	Able to read parts of sentence	0.61 (0.53, 0.70)**	0.94 (0.79, 1.12)
	Cannot read at all	0.72 (0.66, 0.79)**	1.16 (0.96, 1.38)
	Not assessed	0.08 (0.04, 0.13)**	0.70 (0.33, 1.49)
Births in last 5 years	0	1	1
	1	0.99 (0.90, 1.09)	1.10 (0.95, 1.26)
	2+	0.76 (0.67, 0.85)	1.04 (0.87, 1.25)
Number of living children	≤ 2	1	1
	2 to 5	0.88 (0.80, 0.97) *	1.06 (0.89, 1.25)
	>5	0.71 (0.62, 0.80) **	0.75 (0.59, 0.95)*

&, catholic, traditional, and others; *p < 0.05; **p < 0.01.

## Discussion

UHC places a strong emphasis on the importance of providing health services in the community to guarantee that underserved groups have access to care (33). CBHI looks to be an effective strategy for achieving UHC and a promising effort to improve access to healthcare, health outcomes, and social protection in the event of illness (23, 34). Our study finding revealed that nearly four out of 10 (43.2%) reproductive-age women in Ethiopia enrolled in CBHI. This indicates that for more than half of women, the main health expenditure comes from household out-of-pocket payments. Against this backdrop, ensuring access to healthcare services without the risk of financial ruin remained a big challenge that political leaders and the health system in the country are struggling for. Similar findings were reported in Gabon and Indonesia, where 42.8 and 40.0% of women were covered by health insurance (17, 18). On the other hand, the finding is lower than what is reported in Ghana (62.4%). However, this figure is by far higher than study findings in sub-Saharan African countries (8.5%) (18), Nigeria (less than 3%) (20), Ethiopia (4.7%) (15), Kenya (7.0%), and East African countries (7.56%) (19). Evidences in ethiopia revealed that CBHI enrolment depend on households’ willingness to pay, renew their CBHI membership, and enroll in the CBHI program, which highly dependent on the affordability of premium and availability of service (35). Besides, a strong government commitment is essential to achieve UHC. Ethiopia’s government is committed to achieving UHC by improving financial risk protection (23, 36). Yet, this may require innovative approaches like institutionalization of the program and lowering the participation cost through public subsidies or other mechanisms (33).

We found contradicting results regarding the relationship between age and CBHI enrolment. This study showed that the age of the women was a significant factor associated with enrolment in CBHI. Women aged 20–24 years, 25–29 years, and 30–34 years had lower odds of being enrolled in CBHI than their younger counterparts (15–19 years). In this regard, Weldesenbet et al. reported that CBHI enrollment decreased among women aged 20–24, whereas increase for women aged 30–34 years compared to women of the age group of 15–19 years (19). In Nigeria, having health insurance enrollment was significantly higher for women aged 25–34 years and 35 years or older compared with women who are aged 15–24 years (20). Similarly, studies elsewhere reported CBHI membership was positively associated with older age (18, 31, 37, 38). This could be due to a global initiative of universal access to education, allowing younger women to have at least a primary education, up-to-date information, and basic health knowledge. Besides, the interaction between the women’s age and their health-insurance membership may be altered by the other confounding variable. Thus, interventions aimed at increasing the uptake of CBHI among reproductive-age women in Ethiopia need to focus on identifying and targeting certain age groups (20–34 years).

Similar to previous studies (17–19), in this study, women living in rural areas had lower enrollment in CBHI than urban residents. This maybe due to women living in urban areas had good information access and the economic advantage to engage in CBHI. Weldesenbet et al. posit that less information access and media exposure among rural dwellers decreased their enrollment in health insurance schemes (19). Amu et al. explained this, most health facilities cited in urban areas and healthcare systems also mostly focused on urban areas than rural areas. Access to healthcare is thus, usually a challenge for reproductive-age women, especially in pregnancy and childbirth situations. This was the most persistent challenge to UHC including the CBHI schemes (18). As the CBHI aims to reach a large segment of the rural population, the government of Ethiopia needs to emphasize reproductive-age women living in rural areas.

Our study found a regional variation in women’s enrollment in CBHI. Compared to women in the Tigray region, those in the Afar, Amhara, and Oromia regions, Addis Ababa and Dire Dawa cities had higher enrollment in CBHI. This could be due to these regions being relatively urban and health facilities being more accessible. Whereas, women in the Somali, Benishangul, SNNPR, and Gambela regions had lower enrollment in CBHI as compared to those in the Tigray region. Another study also reported the highest CBHI coverage in Amhara, Tigray, southeast Benishangul and the western part of Afar regions, whereas comparatively modest coverage in Addis Ababa (31). According to Kebede et al., the disparity may result from regional variations in the availability of tertiary care and premium payment options. In Amhara and Tigray, CBHI members may visit any public hospital within the region (31).

Our study also found women with a higher education level were more likely to be enrolled in CBHI than non-educated women. Similar findings were reported by studies conducted in sub-Saharan African countries, east African countries, Ethiopia, Nigeria and Indonesia (15, 17–20). A possible reason is that educated women have more knowledge about the advantages of CBHI and make informed choices about their health including purchasing health insurance. In addition, education is most likely associated with access to information, employment, and high income which foster CBHI membership. Uneducated women may not know the advantages of CBHI.

In this study, women affiliated with Protestant religions were 28% less likely than those affiliated with Orthodox religions to be enrolled in CBHI. Women affiliated with Muslim and other religions (Catholic, traditional, and others) were more likely enrolled in CBHI than those affiliated with Orthodox religion. The finding of this study is in contrast to a study done in Nigeria (20). In studies done in Northwest Ethiopia religion is not a significant predictor of enrollment in CBHI (39, 40). This could be because the study populations have different socioeconomic and cultural backgrounds. This may be due to other confounding factors that require further investigation.

In this study, women with larger family sizes were less likely to be enrolled in CBHI than women with smaller family sizes. This study finding is similar with the finding of a study on CBHI utilization among rural households in Akaki District, Ethiopia (41) which indicates the significance of family size. However, a similar study conducted in Nigeria demonstrates family size is not a significant variable (20). The discrepancy could be due to differences in the study setting and socio-demographic characteristics.

Being female household heads were 19% less likely to enroll in CBHI. This finding is consistent with a similar study done in Ethiopia reported by Tadesse G., which revealed that female-headed households were 63% less likely to enrolled in CBHI compared to the male headed housholds (42). Women who had a large number of living children were less likely to enroll in CBHI. In contrast, studies in revealed people with large family members were more likely to pay for the CBHI scheme than those with few family members (35, 43). This could be attributed to the financial burdens that women with large family size had to enroll and renew their CBHI membership.

## Strengths and limitations

One of the key strengths of our study is that we used the current population-based national representative DHS data with a large sample size for the analysis. This ensures that our findings reflect the current realities in the nation. Moreover, the data collection of the surveys used for the studies featured high standard methodological procedures. However, this study is not free from limitations. The cross-sectional design adopted in collecting the DHS data limits our ability to make any causal inferences among the variables studied. Since this study was a secondary analysis of DHS data, some important variables like the current health condition, chronic illness, cost of health care, and health service experience of CBHI members were not available. Given the retrospective nature of the questionnaire-based survey, recall bias might be the other limitation of this study.

## Conclusion

The overall CBHI enrollment among reproductive-age women in Ethiopia was relatively low. To achieve SDGs, increasing reporductiva age women’s CBHI enrollment in all areas of the country needs to be ensured. Such schemes are still able to provide valuable benefits for reproductive-age women to access healthcare. The study also revealed that reproductive-age women aged 20 and 34 years, rural residents, and women living in the Somali, BenshanguleGumuz, SNNP, and Gambella regions, women affiliated with Protestant religion, women with large family size, female-headed households, and those with more than five living children were associated with low CBHI enrolment. Thus, improving reproductive-age women’s understanding of the CBHI system developing their trust in it, and enabling them recognize its valuable benefits require an emphasis of healthcare providers, lower level policy makers, and operational staff who mobilize the development of CBHI. The national, regional, and local officals, policymakers, NGOs, program planners, and other supporting organizations working on improving health insurance enrollment of reproductive age women need to create awareness and support them based on these significant factors.

## Data availability statement

Publicly available datasets were analyzed in this study. The raw data used in this study can be accessed from the DHS website: https://dhsprogram.com/.

## Ethics statement

This study used publicly available EMDHS data. The survey protocol was reviewed and approved by the Federal Democratic Republic of Ethiopia Ministry of Science and Technology and the Institutional Review Board of ICF International. Permission was obtained from the Measure DHS program to access and analyze the data. During EMDHS data collection, informed consent was taken from each participant, and all identifiers were removed and the confidentiality of the information was maintained. As a result, ethical approval and consent to participate in the study are not applicable.

## Author contributions

SH conceived the study design. TB, GG, TD, and BW carried out the statistical analysis. SH, TB, GG, TD, and BW conducted the literature review. SH and TB wrote draft manuscript. GG, TD, and BW reviewed and commented the draft manuscript. All authors contributed to the article and approved the submitted version.

## Conflict of interest

The authors declare that the research was conducted in the absence of any commercial or financial relationships that could be construed as a potential conflict of interest.

## Publisher’s note

All claims expressed in this article are solely those of the authors and do not necessarily represent those of their affiliated organizations, or those of the publisher, the editors and the reviewers. Any product that may be evaluated in this article, or claim that may be made by its manufacturer, is not guaranteed or endorsed by the publisher.

## Footnotes

^1^ http://www.measuredhs.com
